# Cyclodextrins as Anti-inflammatory Agents: Basis, Drugs and Perspectives

**DOI:** 10.3390/biom11091384

**Published:** 2021-09-19

**Authors:** Silvia Lucia Appleton, Silvia Navarro-Orcajada, Francisco Juan Martínez-Navarro, Fabrizio Caldera, José Manuel López-Nicolás, Francesco Trotta, Adrián Matencio

**Affiliations:** 1 Dip. Di Chimica, Università di Torino, via P. Giuria 7, 10125 Torino, Italy; silvialucia.appleton@unito.it (S.L.A.); fabrizio.caldera@unito.it (F.C.); francesco.trotta@unito.it (F.T.); 2 Departamento de Bioquímica y Biología Molecular A, Unidad Docente de Biología, Facultad de Veterinaria, Regional Campus of International Excellence “Campus Mare Nostrum”, Universidad de Murcia, 30100 Murcia, Spain; silvia.navarro6@um.es (S.N.-O.); josemln@um.es (J.M.L.-N.); 3 Department of Developmental and Molecular Biology, Albert Einstein College of Medicine, Bronx, NY 10461, USA; 4 Department of Medicine (Hepatology), Albert Einstein College of Medicine, Bronx, NY 10461, USA

**Keywords:** cyclodextrins, inflammation, drug, review, bioactivity

## Abstract

Inflammation is a biological response of the immune system to harmful stimuli. Importantly, inflammation is also a hallmark of several human diseases such as cancer or diabetes. Novel drugs to treat this response are constantly researched, but the formulation is usually forgotten. Cyclodextrins (CDs) are a well-known excipient for complexing and drug delivery. Anti-inflammatory drugs and bioactive compounds with similar activities have been favored from these CD processes. CDs also illustrate anti-inflammatory activity per se. This review tried to describe the capacities of CDs in this field, and is divided into two parts: Firstly, a short description of the inflammation disease (causes, symptoms, treatment) is explained; secondly, the effects of different CDs alone or forming inclusion complexes with drugs or bioactive compounds are discussed.

## 1. Introduction

Inflammation is a biological process of the immune system in response to harmful stimuli, such as pathogens, damaged cells, or toxic compounds. Inflammation is a hallmark of many diseases including cancer and auto-immune diseases such as psoriasis and rheumatoid arthritis [1]. Immune cells, blood vessels and different immune-mediators are timely orchestrated with the ultimate goal of restore homeostasis by eliminating the initial cause of the damage, achieve resolution, and promote regeneration [2]. Genetic and environmental factors can easily disrupt such precise process interfering with the resolution of inflammation and uncontrolled acute inflammatory response gives place to chronic inflammation, which contributes to a variety of chronic inflammatory conditions [3]. The field has vastly focused on pharmacological ways, not only drugs but also bioactive compounds, to help control and resolve inflammatory responses in multiple scenarios. However, some of them could present low stability or bioavailability needing a formulation improvement. An increase in the drug quantity is sometimes a good solution, but it can cause an increase in adverse side effects. For that reason, the desired solution starts with the increase of stability or bioavailability of the molecule changing the formulation, by for example using cyclodextrins (CDs) [4].

CDs are well-known members of the science community for their uses to solubilize poor-soluble drugs [4,5,6]. Chemically, CDs are truncated cone-shaped oligosaccharides made up of α-(1,4) linked glucose units, obtained by the degradation of starch by the enzyme cyclodextrin glucosyltransferase. The most common CDs are the natural α, β and γ-CD, which contain six, seven and eight glucose units, respectively. The CD ring is a conical cylinder of an amphiphilic nature, with a hydrophilic outer layer (formed by the hydroxyl groups) and a lipophilic cavity [7,8]. When poorly-soluble drugs are complexed with CD, it is created the so-called “inclusion complex” [9,10,11]. To improve the properties of CD monomers, different chemically obtained derivates (e.g., Hydroxylpropyl-β-CD or Methyl-β-CD among others) and polymers have been shown to possess better capacities, such as complexation efficiency or release than natural CDs [12,13,14]. The administration of the drug as complex could increase the efficacy and capacities of the formulation, being an interesting point to improve current treatments. The present review aims to provide a general overview of the use of CDs and their derivatives in the control of inflammation and related processes.

## 2. Inflammation

### 2.1. Basic Principles

The inflammatory response is developed upon activation of Toll-like receptors (TLRs) and other Pattern Recognition Receptors (PRRs) [15,16,17,18,19]. When an insult is detected, epithelial cells and tissue-resident macrophages initiate the inflammatory response. The production of diverse proinflammatory chemokines and cytokines, like TNF-α, IL1B and CXCL8, induce the migration of neutrophils and monocytes, which will differentiate into macrophages, to the inflammation site [17,20]. The release of these mediators and others such as leukotriene B4 (LTB4) and histamine regulate the series of cascade events involved in the inflammatory response including vasodilatation, increased blood vessels permeability, increased expression of endothelial adhesion molecules, swelling and recruitment of immune cells [19,21,22,23]. Inflammation can be classified into acute and chronic phases, based on the severity and duration of the inflammatory response.

In the local acute inflammation, vascular changes and the infiltration of immune cells, mainly neutrophils and macrophages, into the tissues could cause swelling, pain, fever and erythema [24]. When the damage has been cleared, the acute inflammatory process is resolved through the release of pro-resolution and anti-inflammatory molecules, such as protectins, maresins, resolvins and lipoxins [18,25,26,27]. In the case that resolution is not achieved and acute inflammatory response is sustained, a chronic inflammatory response is established and could last several months to years. When inflammation becomes chronic, most characteristics of acute inflammation, such as immune cell accumulation and increased vascularity, continue. However, among the infiltrated cells, in chronic inflammation, there are also lymphocytes [24]. At this stage, neutrophil degranulation promotes lymphocyte activation, which triggers the release of mediators that attract more immune cells to the inflamed tissue. Chronic inflammation could occur when the response is not able to clear a pathogen, in hyper-sensitivity diseases, like autoimmune or allergic diseases, and during long-time exposure to a toxic agent exogenous or endogenous, such as silica or cholesterol, respectively [27].

### 2.2. Inflammatory Diseases

There is a rising incidence in modern societies of cases of systemic chronic inflammation and chronic inflammatory diseases, which are becoming one of the major causes of morbidity and mortality in developed countries [28,29,30]. Systemic chronic inflammation increased the risk of suffering different diseases, like some types of cancer [31], non-alcoholic fatty liver disease [32], metabolic syndrome [33] and type-2 diabetes [34]. C-reactive protein (CRP) is the main chronic inflammation biomarker, it has been associated with an increase in risk for coronary heart disease and cardiovascular disease mortality [35,36]. There is a large range of chronic inflammatory disease, rheumatoid arthritis, diabetes, inflammatory bowel disease, atherosclerosis, Crohn’s disease, allergies, asthma or psoriasis are some examples among them. Due to this situation, in recent years, several studies using different animal models and clinical trials have been focused on finding new molecular targets and compounds to treat these chronic conditions [37,38,39,40].

### 2.3. Anti-Inflammatory Drugs

Corticosteroids are one of the most effective therapy to treat chronic inflammatory diseases. They suppress the multiple inflammatory genes by reversing histone acetylation through binding to glucocorticoid receptors [41]. They are commonly used to treat autoimmune diseases, asthma, psoriasis and rheumatic arthritis. Long-term use of corticosteroids could lead to developing adverse effects, like hypertension, metabolic issues and peptic ulcer [42].

Nonsteroidal anti-inflammatory drugs (NSAIDs) are widely used by the population; they inhibit cyclooxygenase (COX) enzyme activity [43,44,45]. These enzymes are responsible for the productions of different inflammatory mediators as prostaglandins, thromboxanes and prostacyclines [46]. The NSAIDs are highly effective during acute inflammation to reduce the symptoms but they are not curative, and their chronic use could be harmful [47].

Usually, chronic inflammation disease treatments are limited, and their efficacy diminishes along the application time. One of the most powerful is the biologic therapy, which consists of the use of antibodies (suffix -mab) or fusion proteins (suffix -cept) that target specifically the main pro-inflammatory cytokines like TNF-α and IL1-β and block them [48,49]. This approximation usually is used when other treatments are not effective, due to possible side effects [50].

## 3. Cyclodextrins as Agents for Treatment

**Figure 1:**
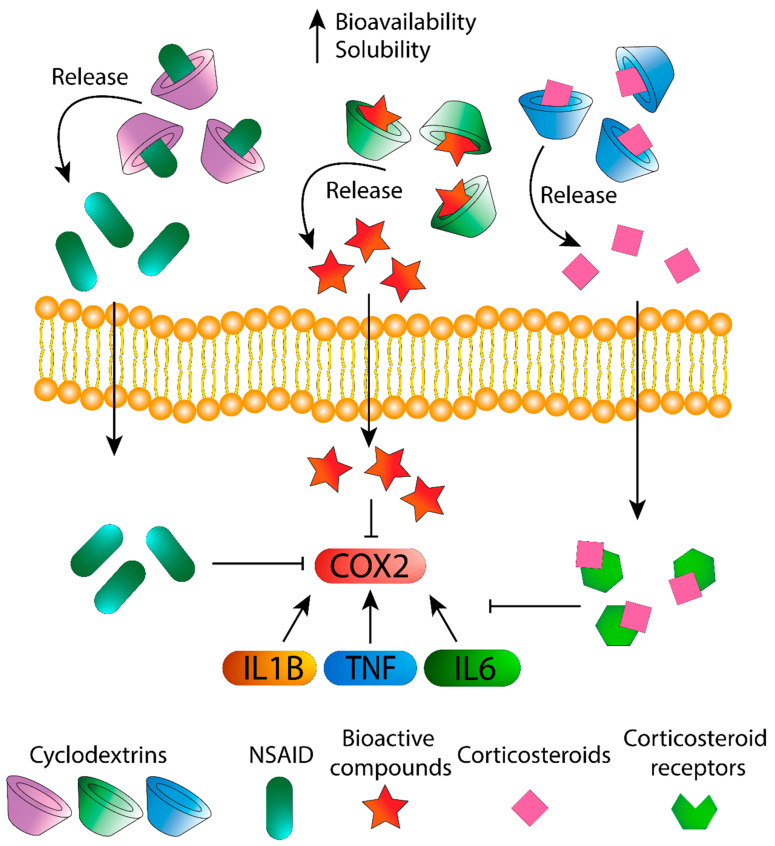
Schematic representation of CD/Corticosteroids and some CD/bioactive compounds complexes against inflammation (depending on the molecule, the pathway could change).

In this section, the effect of CDs alone and complexed with drugs and bioactive compounds against inflammation is discussed (Figure 1).

### 3.1. Drugs

Among the significant number of CD-based formulations that have reached the market [51], the CD-anti-inflammatory drug complexes [52] seem to hold a prominent position.

As far as NSAIDs are concerned, CDs overcame the formulation issues caused by their poor water solubility [53]. The great achievements reached triggered intense research over the years [54,55]. A recent systematic review [55] collects the studies conducted in this last decade (2010-2020) and shows how cyclodextrins have had a huge impact on the formulation of NSAIDs with over 600 articles found in the literature, with 24 different NSAIDs investigated and 60 formulations obtained through combinations of CDs and NSAIDs. It emerges that meloxicam, followed by diclofenac, flurbiprofen, ibuprofen, piroxicam, aceclofenac and oxaprozin is the most studied anti-inflammatory drugs and some of the most recent studies are listed in Table 1 as examples. Among CD types, β-CD and HPβ-CD are frequently used, often combined with meloxicam, piroxicam, ibuprofen and piroxicam, flurbiprofen, respectively.

Successful NSAID-CD complexes have also been patented [63] and commercialized [5]. The first one was introduced in Europe in the 1980s with Brexin, a formulation made up of piroxicam and β-CD [52]. Others are Mobitil (meloxicam/β-CD) produced in Egypt and Flogene (piroxicam/β-CD) in Brazil [5].

Alongside solid dosage forms (suppositories and tablets), there are also liquid CD-based formulations. Indocid Eye drop solution is an HPβ-CD-indomethacin combination and Voltaren Ophtha Eye Drops, an HPγ-CD-diclofenac combination for treating eye inflammation, in which the cyclodextrin is not only a drug solubility enhancer but also a penetration promoter without being harmful to the cornea.

CDs were successful also in the delivery of another class of drugs used for the treatment of inflammation, that is, glucocorticoids [64]. Glucocorticoids are used to restrain inflammation, allergy and immune response and make organ transplants possible [64]. Natural and synthetic steroids are currently used, and dexamethasone is an example of synthetic steroid, which is often used but has low solubility in water. Indeed, the hydrophobicity of glucocorticoids has made their formulation challenging.

At the moment, transdermal is the most common route of administration with glucocorticoid-based formulations in the form of hydrogels. Unfortunately, they can be harmful after continuous exposure to organic co-solvents (e.g., ethanol or DMSO) used for the solubilization of glucocorticoids. CDs have therefore been investigated as a possible alternative to prevent the use of co-solvents.

The attempts made in the delivery of steroids in vitro, preclinical, and clinical studies are collected in the systematic review conducted by Santos et al. [54], which shows the effectiveness of CDs in the delivery of steroids when compared with the free drug in terms of inhibition of inflammatory mediators and edema reduction. For example, a hydrocortisone acetate-βCD complex was studied in vitro and good results were achieved as far as solubility, stability and drug release are concerned [65]. Dexamethasone combined with HPβ-CD was tested in vivo on rabbits affected by uveitis and improved the inflammation condition [66]. Alpha-methyl prednisolone complexed with a CD polymer drastically reduced the arthritis scores and paw edema compared with pure drug [67].

Prednisolone 21-hemisuccinate in α-CD was tested on rats with inflammatory bowel disease and colon damage score and myeloperoxidase activity were reduced [68,69]. Dexamethasone has been investigated in clinical studies for the treatment of diabetic macular edema and cataract with successful results. The CDs selected were γ-CD and HPβ-CD, administered topically (ophthalmic route) [70,71].

The aforementioned studies are grouped in the following Table 2.

In-depth knowledge of CDs, as well as the efforts for the optimization of CD formulations, has led to the development of supramolecular structures (e.g., self-assembled systems, cross-linked polymers, drug-conjugates), which improved the limits of single CDs and transformed them into advanced drug delivery systems [51,72].

Indeed, the use of CDs is limited as guest molecules need to interact and fit inside the CD cavity, as a consequence hydrophilic or high molecular weight drugs are not suitable for complexation with CDs. One of the strategies proposed is reacting native cyclodextrins with a cross-linking agent to form insoluble polymers, called nanosponges (NSs) [13]. They have a peculiar cage-like structure formed by cyclodextrins connected by nanochannels, which can be modulated and consequently affect the inclusion capacity. There is a broad number of molecules that can be incorporated inside NSs as there are many interaction sites available for the formation of inclusion and non-inclusion complexes [73]. In particular, the hydrophobic CD cavities with hydrophilic nanochannels of the polymeric network around them permit interaction with drugs having different degrees of lipophilicity and structures [74], including NSAIDs and steroids.

Cavalli et al. tested the capacity of cyclodextrin based nanosponges (CD-NSs) to load lipophilic drugs, i.e., dexamethasone and flurbiprofen. The NS not only loaded the drugs but also released them in a sustained manner [75]. Shende et al. [53] compared CD with CD-NSs for the delivery of meloxicam to understand the improvements that a NS could provide. The low aqueous solubility of meloxicam was enhanced when encapsulated inside NSs and improved compared to CDs. Additionally, the zeta potential was highly negative in the NS formulation, thus suggesting that it was stable. It was then tested in vitro and in vivo and an improvement in rat paw edemas was achieved. The authors thus concluded that NS could be promising drug delivery systems in which the release of meloxicam is controlled to obtain the maximum anti-inflammatory effect.

In another study, β-cyclodextrin nanosponges were used to deliver naproxen. Not only the NS was able to carry the drug but also to prolong and modulate its release being pH-sensitive [76]. Additionally, ibuprofen has been the focus of a few studies [77,78], in which the objective was to understand the diffusion properties of the drug across the NS matrix to optimize polymer synthesis, thus opening the way to the design of drug delivery systems with the desired drug release properties.

### 3.2. Anti-Inflammatory Bioactive Compounds

Bioactive compounds from natural sources could be an appealing alternative to synthetic drugs when designing a CD-formulation with anti-inflammatory activity (Table 3). Some example of these compounds can include stilbenes (resveratrol), flavanones (hesperidin and alpinetin), flavanolols (ampelopsin and dihydromyricetin), flavones (baicalein, apigenin, chrysin, luteolinnaringenin and naringin), flavonols (galangin), flavonoid glycosides (diosmin and rutin) and terpenes (betulin and zerumbone) [79].

Resveratrol is the most-studied stilbene found in wine and grapes and possesses a limited solubility in water. Lin et al. (2020) [80] have successfully integrated it in HPβ-CD/PVP electrospun nanofibers to overcome that problem and tested them in HaCAT keratinocytes. Treated cells suppressed Particle Matter (PM)-induced expression of inflammatory proteins, COX-2 and matrix metalloproteinase-9 (MMP-9) at a dose of 20 µM, indicating that nanofibers retained the anti-inflammatory activity of the compound. Lim et al. 2020 [81] evaluated the anti-inflammatory effect of pterostilbene, an analogue of resveratrol, complexed with HPβ-CD in RAW 264.7 macrophage cells treated with *Fusobacterium nucleatum*. The results showed that the inclusion complexes inhibit NF-κB activity and decrease the expression of TNF-α, IL-1β and IL-6, while cytokine IL-10 was not affected.

The isoflavone genistein was encapsulated in HPβ-CD in order to improve its solubility and bioavailability for atopic dermatitis applications. These complexes were found to down-regulate mRNA expression of anti-inflammatory cytokines (IL-1α, IL-1β, IL-6 and TNF-α) below the concentration that caused cytotoxicity (10 µg/mL) [82]. Another flavonoid, baicalein, enhanced its solubility and stability after encapsulation in HPβ-CD, and the inclusion complexes were used to design a thermosensitive hydrogel formulation that alleviated inflammation in animals with cervicitis [83].

**Table 3:** Summary of the most relevant bioactive compounds and their effects.

Type of CD	Bioactive Compound	Natural Source	Effect	Reference
HPβ-CD/PVP	Resveratrol	Wine, grapes and blackberries	Induced expression of inflammatory proteins, COX-2 and MMP-9	[80]
HPβ-CD	Genistein	Soybeans	Down-regulate mRNA expression of anti-inflammatory cytokines	[82]
HPβ-, Mβ- and HPγ-CD	Curcumin	Tumeric	Treating inflammatory disorders	[84,85]
HPβ- and β-CD	Citral	Lemons and oranges	Reduced total leukocyte migration into the pleural cavity and TNF-α levels	[86]
HPβ-CD	Naringenin	Grapefruits and oranges	Reduced TNF- α levels	[87]
β-CD	Carvacrol	Oregano essential oil	Decrease level of IL-1β, IL-6, MIP-2 and TNF-α and higher of IL-10	[88]
α-CD	Moringin	Moringa	Down-regulated pro-inflammatory cytokines TNF-α and IL-1β	[89]

Curcumin, a natural coloring from turmeric, decreased the extent and severity of the injury of the large intestine in synthetic dextran sulfate solution (DSS)-induced experimental colitis model in Sprague Dawley rats when complexed with HPβ-CD and Mβ-CD at 1:1 and 1:2 stoichiometries [84]. Additionally, the use of water-soluble curcumin with HPβ-CD or HPγ-CD to manufacture a medicament for treating inflammatory disorders such as rheumatoid arthritis, psoriasis, ulcerative colitis and Crohn’s disease, has been patented [85]. Along with cyclodextrins, Sawant et al. (2014) developed PEG-coated zinc ferrite nanoparticles with curcumin/β-CD complexes that protect erythrocyte membrane against lysis induced hypotonic solution. As human red blood cells membranes are similar to lysosomal membranes, they considered the prevention of hypotonicity-induced erythrocyte membrane lysis as a measure of anti-inflammatory activity.

Ellagic acid complexed with β-CD was also able to protect erythrocyte membrane from lysis induced by heat and hypotonicity, as well as protect albumin from denaturation, demonstrating that the encapsulation improves the anti-inflammatory effects of the bioactive compound [90]. Although the authors prepared 1:2 complexes with CDs, it has been described that ellagic acid can form 1:1 complexes with CDs in the presence of borax [91].

The inhibition of COX or LOX by different betalains, a family of bioactive compounds with interesting bioactivities was recently published [92]. The problems of stability of these compounds can be solved with CDs when the inclusion complex is formed [93].

Some natural compounds from citric fruits have also been encapsulated in cyclodextrins and their anti-inflammatory activity evaluated in animal models. This is the case of citral/β-CD and citral/HPβ-CD complexes which reduced total leukocyte migration into the pleural cavity and TNF-α levels in Swiss mice fed with 100 mg/kg [86]; naringenin/HPβ-CD complexes which also decreased TNF-α levels and showed similar activity to that achieved with naringenin as supplied but administering only one-fifth of its dose [87]; and limonin/β-CD and limonin/γ-CD complexes which reduced the volume of paw edema in Wistar rats fed with 0.12 mg/kg, as well as, improved articular function by the decrease in the degree of bone resorption, soft tissue swelling and osteophyte formation [94].

Neochlorogenic acid, a phenolic compound, has been demonstrated to inhibit microglia activation and pro-inflammatory responses in the brain (inhibition of TNF-α and IL-1β and block of phosphorylated NFκB p65 and p38) [95] and recently, its complexation in natural and modified cyclodextrins have been evaluated at different pH [96].

Terpenoids complexes like carvacrol with β-CD have been tested in animal models for anti-inflammatory effects revealing a reduction in hyperalgesia and in spontaneous and palpation-induced nociception in mice with a tumor on the hind paw [97], and a decrease in muscle tissue myeloperoxidase activity (MPO) and edema after carrageenan treatment in rats [88]. In the last study, the authors also observed a lower level of IL-1β, IL-6, MIP-2 and TNF-α and higher of IL-10 as compared to the vehicle group. A greater reduction of paw edema formation induced by carrageenan was observed in mice treated with the monoterpene p-cymene complexed with β-CD in comparison with p-cymene alone [98]. Furthermore, pedunculoside was embedded into a β-CD polymer with the cross-linking agent epichlorohydrin. The resulting complex exhibited low toxicity and acted more effectively on mice ear edema than the free triterpene, possibly because the improvement of aqueous solubility after encapsulation contributes to the absorption [99]. Moreover, complexation with β-CD improved the solubility and stability of linalool, leading to an enhancement in its antinociceptive and analgesic effects by the reduction of total leukocyte migration and TNF-α levels in peritoneal fluid [100], and a significant reduction of hyperalgesia on chronic non-inflammatory muscle pain model [101].

Moringin, the main isothiocyanate from *Moringa oleifera* seeds, was complexed in α-CD to increase solubility and stability. The complexes down-regulated pro-inflammatory cytokines TNF-α and IL-1β in LPS activated macrophages cells by preventing IκB-α phosphorylation, suppression of Akt and p38 phosphorylation and translocation of NF-κB [89]. Additionally, modulation of oxidative stress was observed.

The alkaloid berberine was also described to have an anti-inflammatory effect and its complexation in natural β-CD and α-CD was achieved, revealing that the latter provided a better yield [102]. Coumestrol, a plant estrogen, was found to induce cell proliferation and migration in the inflicted wound in Wistar rats after being supplied in HPβ-CD complexes [103].

*Centella asiatica* extract is rich in asiaticoside, a bioactive compound that is described to promote the synthesis of collagen and acidic mycopolysaccharides and inhibit the inflammatory phase in wound healing that causes hypertrophic scars and keloids. Srichana et al. (2016) [104] developed a topical spray formulation with this extract and HPβ-CD that was non-irritating in the rat model and was able to completely heal an excision wound after 14 days, faster than the control. Moreover, *Terminalia sericea* extract with 86 % of sericoside was encapsulated in γ-CD, HPγ-CD, HPβ-CD and Mβ-CD to improve its solubility, and the oil/water formulation containing the complexes results in a 2.6-fold higher percutaneous penetration of sericoside in excised pig skin compared with pure extract [105].

Brazilian green propolis extract rich in artepillin C and also p-coumaric acid, baccharin, drupanin and cinnamic acid, was complexed with γ-CD and orally administered to mice [106]. A down-regulation of mRNA levels of TNF-α, decrease in gene expression of serum amyloid P and induction of hepatic ferritin gene expression were observed, while endogenous antioxidant activity was not affected.

Complexes of basil essential oil/β-CD aimed to enhance bioavailability, also inhibit granuloma formation and leukocyte recruitment to the peritoneal cavity, and prevent paw edema formation by the decrease in vascular permeability [107]. Pinheiro et al. (2017) [108] develop β-CD and HPβ-CD complexes with *Copaiferamultijuga* oleoresin that retained anti-inflammatory activity measured by carrageenan-induced paw edema test. Moreover, electrospun nanofibers of HPβ-CD and plai oil from *Zingiber cassumunar* Roxb. have been proposed as an alternative topical application due to the anti-inflammatory activity of this essential oil [109].

### 3.3. CD as Active Agents in Inflammatory Diseases

The capacity of CDs to complex different agents can be used to manage inflammation directly, as occurs with other diseases such as Niemann Pick or neurological diseases [110,111,112]. A case is the atherosclerosis, where the cholesterol accumulation in veins starts the recruitment of macrophages and the inflammation response [113]. HPβ-CD was able to treat atherosclerosis not only by increasing the efflux of cholesterol [114] but also through macrophage reprogramming [115] by the LXR-mediated signaling pathway; cholesterol efflux was increased as a result of ABCA1 and ABCG1 upregulation, which was corroborated in another recent study where this CD reduced the levels of plasma triglycerides and inflammatory cytokines, and also increased the level of plasma HDL-cholesterol. HPβ-CD demonstrated to interact with cholesterol crystal reducing the IGs deposition and the activation of complement activation as measured by terminal complement activation and lowered specific receptor expression on monocytes [116]. Even, the administration of HPβ-CD reduced the level of pro-inflammatory cytokines (IL-1α, TNF or IL-6 among others). This effect was tested with another deposition such as monosodium urate crystal without effect, suggesting a specific effect against cholesterol crystals. Pilely et al., in 2019 discovered that α-CD inhibits cholesterol crystal-induced complement-mediated inflammation as HPβ-CD [117].

## 4. Discussion

In the light of the considerations made above, it is clear that CDs respond to the need to optimize treatments that already exist, making them more effective, stable and safe. Encapsulation of CDs has been demonstrated to be efficient in solving these kind of issues [8,118] while maintaining or even increasing the biological activity of these agents because they act as multifunctional excipients in that they are solubility enhancers, prevent drug–drug and drug–additive interactions within a formulation, eliminate unpleasant smells or tastes and reduce side effects [5,53,119,120]. Even their own activity as active drugs could promote possible synergetic capacities [116]. In fact, they can be found in numerous pharmaceutical forms, e.g., tablets, suppositories, droplets and spray.

Last but not least, they can be used to develop advanced drug delivery systems that go beyond the limits of single cyclodextrins. As a perspective, all these properties have been found to be ideal for the formulation of anti-inflammatory drugs and bioactive compounds. In addition, novel CDs are created to improve the possible limitations, being able to complex not only drugs but also heavier molecules such as proteins. Bearing the above features in mind and judging by the number of studies conducted, they are certainly in the limelight, so research on this topic is far from over.

In conclusion, this review emphasizes the role of CDs for inflammation treatment. They have been used to improve the release and bioavailability of different approved drugs. Similar studies have been carried out with bioactive compounds where the managing of inflammation responses such as oxidation and the expression of pro-inflammatory cytokines were reduced. On the other hand, CDs have demonstrated their own capacities with cholesterol mediated inflammation processes, managing the complement activation. In summary, this review indicates a possible combinatorial effect where the drug is complexed with CDs against inflammation. In simple terms, the application of CD lays the groundwork for future progress opening up a new realm of other advanced applications expected to arise soon.

## Figures and Tables

**Table 1:** Examples of recent studies conducted on CD-NSAID complexes.

Type of CD	NSAID	Objective of the study	Reference
β-CD	Meloxicam	In vitro evaluation for periodontitis treatment	[56]
HP-β-CD	Diclofenac	Clinical evaluation of the pharmacokinetics of diclofenac in patients with mild or moderate renal insufficiency or mild hepatic impairment	[57]
HP-β-CD	Flurbiprofen	In vitro drug release, mucoadhesion, and irritation potential study for ocular delivery	[58]
β-CD	Ibuprofen	In vitro evaluation of ibuprofen properties in metal organic frameworks	[59]
β-CD	Piroxicam	In vivo evaluation of the analgesic activity and anti-ulcerogenic potential of piroxicam in rats	[60]
β-CD	Aceclofenac	Ex vivo evaluation of stability and transdermal delivery to the inflammatory sites in osteoarthritis	[61]
β-CD	Oxaprozin	In vivo evaluation of the anti-inflammatory activity on adjuvant-induced arthritis in rats	[62]

**Table 2:** Studies conducted on CD-steroid complexes.

Type of CD	Steroid	Objective of the Study	Reference
β-CD	Hydrocortisone acetate	Determination of the anti-inflammatory effect on LPS-stimulated RAW267	[65]
HPβ-CD	Dexamethasone	Determination of the anti-inflammatory effect on rabbits affected by uveitis	[66]
γ-CD	Dexamethasone	Clinical assessment of the anti-inflammatory effect on diabetic macular edema and cataract	[70,71]
HPβ-CD	Dexamethasone	Clinical assessment of the anti-inflammatory effect on diabetic macular edema and cataract	[70,71]
α-CD	Prednisolone 21-hemisuccinate	Determination of the anti-inflammatory effect on rats with inflammatory bowel disease	[68,69]

## Abbreviations

CD	cyclodextrin
HPβ-CD	2-hydroxypropyl β-cyclodextrin
HPγ-CD	2-hydroxypropyl γ-cyclodextrin
Mβ-CD	methyl β-cyclodextrin
PVP	polyvinylpyrrolidone
PEG	polyethylene glycol
TNF-α	tumoral necrosis factor α
IL	interleukin
MPO	myeloperoxidase
MIP-2	macrophage inflammatory protein
NF-κB	nuclear factor κB
LPS	Lipopolysaccharide
TLR	Toll-like receptor
PRRs	Pattern Recognition Receptor
CRP	C-reactive protein
NSAID	Nonsteroidal anti-inflammatory drug
LOX	Lipoxygenase
COX	Cyclooxygenase
